# Temperature-driven response reversibility and short-term quasi-acclimation of *Daphnia magna*

**DOI:** 10.1371/journal.pone.0209705

**Published:** 2018-12-21

**Authors:** Mara F. Müller, Jordi Colomer, Teresa Serra

**Affiliations:** Department of Physics, University of Girona, Girona, Spain; VIT University, INDIA

## Abstract

Analysing the effect water temperature has on *Daphnia magna* is essential in anticipating the impact climate change will have on this freshwater zooplanktonic keystone species. While many authors have followed this line of research, few have covered an extensive temperature range or complex temperature change scenarios. Global warming is mostly associated with increased extreme temperature events, such as heat waves, as well as earlier and more intense thermal stratification. Both of these events may directly influence *D*. *magna* fitness, especially in those populations performing diel vertical migration (DVM). We analysed the effect water temperatures, ranging from 11 to 29°C, have on the filtration capacity (FC) of *D*. *magna*, to anticipate the effects of acclimation, temperature change rate (TCR) and potential reversibility of responses to such conditions. Results show that sudden temperature changes have an immediate negative impact on the FC of *D*. *magna* and is more severe at higher temperatures and higher TCRs. However, *D*. *magna* individuals have shown themselves to be capable of quasi-acclimating to temperatures ranging from 11 to 25°C in around a week and achieving much higher FCs; albeit never reaching the optimal FC achieved at 20°C. That said, 29°C is lethal for *D*. *magna* individuals within approximately five days. Finally, non-optimal temperature acclimated individuals can recover maximal FC within 2–4 days of the optimal long-term acclimation temperature (20°C) being re-established, thus proving temperature responses to be reversible.

## Introduction

Temperature is a key factor determining the life cycle and fitness of all organisms. In the range of normal activity, metabolic rates of living organisms increase exponentially with temperature [1]. However, there is a high variability amongst species when it comes to a range of tolerable temperatures, as well as the optimal temperature for growth, and this greatly depends on whether species are endothermic or ectothermic.

*Daphnia magna* is a key zooplanktonic organism found in many freshwater systems, such as shallow lakes and ponds, which experience high daily and seasonal water temperature fluctuations [2]. *D*. *magna* is known to cause the ‘clear-water phase’ of a lake, when the phytoplankton bloom over where they usually feed, is followed by a growth in *Daphnia* populations, causing the phytoplankton populations to reach a minimum concentration [3–6]. Temperature has been proven to have a decisive effect on the timing and magnitude of the clear-water lake phase in the spring [7–11]. When the water temperature rises, ectothermic cladocerans like *D*. *magna*, present a significant increase in their metabolic rate. As a consequence, they show higher particle uptake rates [12–17] as well as a decrease in survival time and an increase in growth rate [18,19], although this also results in reduced body sizes [16,20–22], probably as a cost of the increased metabolic rates [16]. A decrease in water temperature, however, causes a decrease in metabolic rate, thus resulting in lower particle uptake rates [12–15], reduced growth rate [19] and lower metabolic costs and, therefore, greater body sizes [20]. In terms of environmental conditions, Shelford [23] defined the law of tolerance, (later extended by Schwerdtfeger [24]), by describing three tolerance ranges: optimal, pejus and pessimum. In the optimum temperature range, the increase in aerobic metabolism is maximal, resulting in an optimal fitness [25]. In the suboptimal warm or cold temperature ranges, known as pejus (pejus = worsening), individuals are able to survive, although present reduced fitness, since the physiological processes are impeded in such conditions [26,27]. Finally, in the pessimum range, organisms turn to anaerobic metabolism to cope with oxygen demand, which allows only short-term survival [25]. Many authors have studied the upper thermal limits of *D*. *magna* in the pessimum range, where filtration capacity (FC) [14] and growth rate [22] decrease rapidly because the oxygen transport mechanisms cannot cover the increasing oxygen demand [25,28]. That said, similar effects occur at the low pessimum temperature range, although the lack of oxygen supply is not explained by the misfunction of the transport mechanisms, but rather by the cessation of the muscular activity needed for the correct supply of oxygen [29], leading to a decrease in the PO_2_ in the blood [25]. However, the optimum thermal tolerance window depends a great deal on the acclimation temperature, which promotes significant shifts in the preferred temperatures of *D*. *magna* [25,29], as well as their FC [30]. McMahon [14] found the maximum FC of *D*. *magna* was 24°C, whereas other authors, all of whom had performed a previous long-term acclimation at 20°C, fixed the optimal temperature as being between 20 and 21°C [2,21,31]. Moreover, mortality quantifications show that above 26°C, the survival of *D*. *magna* decreases drastically [12,21]. However, few studies cover an extensive temperature range [15] that includes both the low and high pessimum ranges.

The overall temperature increase in freshwater systems is correlated to the increase in the global mean surface temperature attributable to climate change [32]. This global warming is mostly associated with high-frequency extreme temperature events, in particular more frequent and longer heat waves [32,33], leading to physiological stress in most species [34]. Heat tolerance and thermal adaptation has been studied to predict organisms’ responses to climate change [35–37]. Although thermal acclimated *D*. *magna* individuals present higher heat tolerance [18,25,28,29,38], little is known about how long it takes these organisms to acclimate to the speed of the change in temperature, the severity of extreme events inflicted by climate change [39] and/or what the costs are. Acclimation and sensitivity to short-term temperature changes are critical parameters to the differential plastic and evolutionary responses of *D*. *magna* [40]. Thanks to the strong phenotypic plasticity featured by *D*. *magna*, these organisms are capable of responding rather well to quick and drastic changes in their environment, such as temperature [38,40] or predation [41], with response times ranging from seconds to generations. Since the response to certain environmental conditions may be associated with costs, it is potentially beneficial for individuals to reverse changes in phenotype once the inducing factor has diminished. This is done through reversible responses [42]. Mikulski et al. [41] proved that a reaction to a predation threat initiated by *D*. *magna* can be reversed when the inducing factor is absent for at least four instars.

*D*. *magna*, as well as many other zooplanktonic species, must deal with temperature changes when performing diel vertical migration (DVM) in lakes where they move downwards at dawn and upwards at sunset, mainly to avoid predators [43–45]. This strategical behaviour implies that individuals have to handle rapid and significant temperature gradients as they move through the water column, thus tackling reversed temperature changes of around 10°C [19,46]. The associated temporal changes in temperature are up to 6–30°C/day, if they are to cross the thermocline [47], which is associated with several metabolic costs [19,45,48]. Increased water temperature due to climate change has a direct influence on thermal stratification [49]. Warmer years cause an increase in lake water surface temperatures leading to an earlier and intensified summer stratification. This effect has been proven to cause an advance in the stratified period of up to 20 days and lengthened by 2–3 weeks in several lakes in Europe and North America [49]. Therefore, the temperature gradient and the associated metabolic costs involved for *D*. *magna* individuals performing DVM, may significantly increase due to climate change.

Following this line of research, our study quantifies the effect water temperature has on *D*. *magna* filtration, to anticipate the effects of acclimation, rate of temperature changes and potential reversibility of responses to temperature conditions between 11 and 29°C and to four different temperature rate changes.

## Methods

*Daphnia magna* individuals were originally obtained from the EDAR wastewater treatment plant in Empuriabrava, (Castelló d’Empúries, Spain) and have been maintained for two years in the laboratory at the University of Girona. The University of Girona, through private Contract CCB2016 was allowed to extract the individuals at the WWT Plant. The individuals were cultured in 40 L containers with calcium rich water (35.7 mg/L), following Riessen et al. [50], at a temperature of 20 ± 0.5°C, with a natural daylight photoperiod and a continuous air supply. All the tests, protocols and analysis with *Daphnia magna* were carried out aligning with the international ‘OECD/OCDE Guidelines for the Testing’ and the ‘Protocol of sampling and laboratory tests of invertebrates’ code ML-L-I-2013 of the Ministerio de Agricultura, Alimentación y Medio Ambiente of the Spanish Government. Feeding was carried out three times a week (Monday, Wednesday and Friday) with dry spirulina powder and Baker’s yeast (*Saccharomyces cerevisiae*). One third of the water from the culture was renewed twice a month.

**Fig 1:**
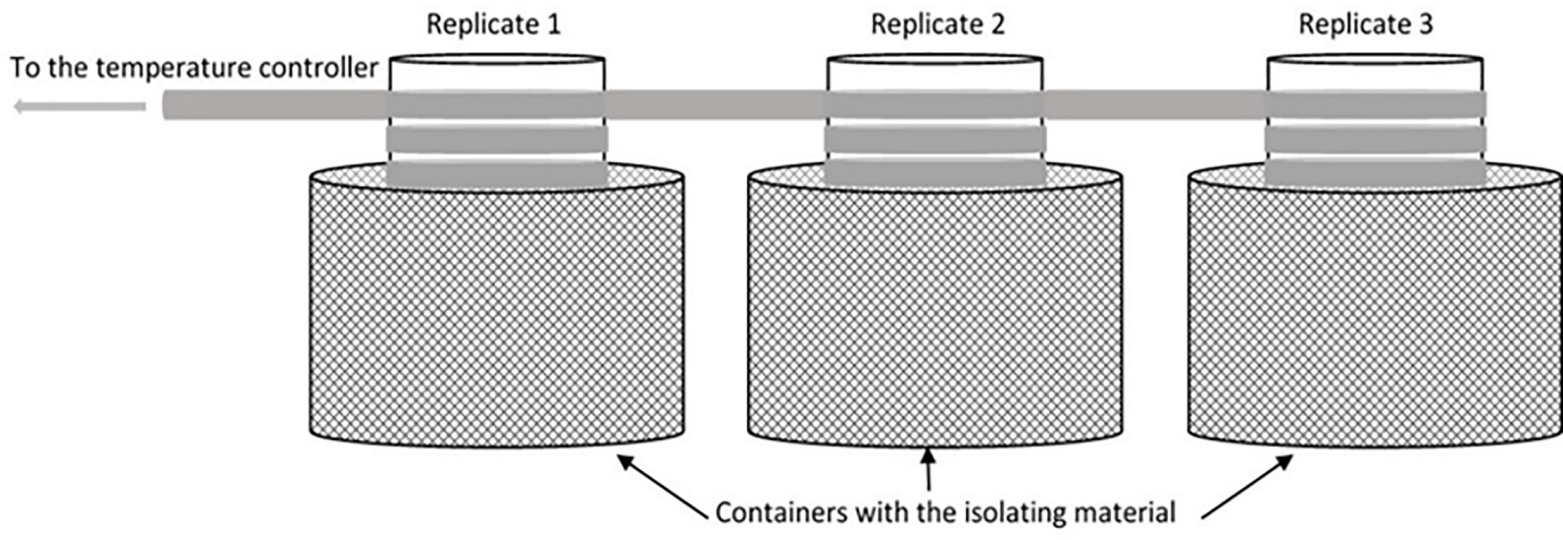
Sketch of the experimental set-up, with three replicates for each experiment. The three replicates were containers where water temperatures were reproduced. The containers were isolated from the exterior, while a temperature controller supplied water through tubes to maintain the experimental temperature in the containers.

Each experiment was performed in Plexiglas containers filled with 1.9 L of bottled mineral water and 100 mL of spirulina suspension. The mineral water matched the characteristics of the water in the containers where daphnids first grew: Ca: 35.7 mg/L, Na: 8.3 mg/L, SiO_2_: 27.1 mg/L, and HCO^−3^: 165 mg/L. The dissolved oxygen concentration was 8.7 mg/L at 21°C and varied with temperature changes. For experiments at a constant temperature, the oxygen did not change at all during the experiments. To prepare the spirulina suspension, 1 g of spirulina powder was diluted in 1 L bottled mineral water, mixed for 60 s at 100 rpm and left for 1 h to let any large spirulina particles settle. The supernatant was used as the spirulina suspension for the experiments. The spirulina particle size distribution in the suspension was analysed using the Lisst-100x (Sequoia Inc.) and FC of *Daphnia magna* was calculated (see S1 Appendix). Once the spirulina suspension was added to the bottled mineral water, 100 *D*. *magna* individuals that were initially obtained from a single brood from the EDAR wastewater treatment plant in Empuriabrava, were carefully introduced into the Plexiglas containers, obtaining a final concentration of 50 individuals L^-1^ (hereafter ind L^-1^), which is the minimum concentration needed to ensure adequate particle removal efficiency (about 30%) [4,51]. *D*. *magna* individuals for the experiments were collected from the laboratory cultures using a mesh with a spacing of 1.5 mm. The mean body length of the individuals was analysed from a video recording of 10 individuals using the ImageJ software and were 1.8 ± 0.2 mm. Temperature changes were performed using a PolyScience temperature controller connected to a silicone tube carrying a continuous and unidirectional flow of water at the corresponding temperature (Fig 1). This tube surrounded the Plexiglas containers placed in chambers with isolating material, to maintain the water at the desired temperature. Each experiment consisted of two replicates and one additional culture maintained at the same experimental conditions in order to have individuals to replace the dead ones.

The range of temperatures analysed was between 11 and 29°C, which are the maximum and minimum water temperatures of the water systems daphnids can encounter in northern and central Spain. Similar lakes in Israel, Iran and Italy are reported to present maximum water temperatures around 30°C [38]. Firstly, sixteen experiments were performed to examine the effect that temporal variation had on the temperature change (Table 1). Four temperature change rates (TCR) were studied (V1, V2, V3 and V4). They were chosen from reported diel vertical migrations of *Daphnia magna* in lakes [19,46]. Eight experiments were performed at a temperature variation of 5°C, by raising the temperature from 20 to 25°C or by lowering it from 20 to 15°C, with TCRs of 1.6 (V1), 5 (V2), 10 (V3) and 20 (V4)°C/day (Table 1). Eight additional experiments were performed at a temperature variation of 9°C, by increasing the temperature from 20 to 29°C or by decreasing it from 20 to 11°C, with TCRs of 1.8 (V1’), 4.5 (V2’), 9 (V3’) and 18 (V4’)°C/day (Table 1). Secondly, twelve more experiments were carried out by reverting the temperature alterations of both the ± 5°C and ± 9°C temporal variations, at the TCRs of V2, V3 and V4, and V2’, V3’ and V4’ (Table 1). *D*. *magna* cultures in the Plexiglas containers were left for the time required to reach the final temperature at the indicated rate. Once the temperature change had occurred, the water was renewed, and new spirulina suspension was added to analyse the time evolution of the suspended spirulina particle concentration under the different experimental conditions after 4 hours. *D*. *magna* survival was evaluated by counting the individuals after 24 hours of exposure to the final temperature.

**Table 1:** Temperature change rates for the acclimation and reversible acclimation experiments.

V(°C/day)				V’(°C/day)				
1.6	5	10	20	1.8	4.5	9	18	
20°C →25°C				20°C →29°C				Acclimation
20°C →15°C				20°C →11°C				
	25°C →20°C				29°C →20°C			Reversible acclimation
	15°C →20°C				11°C →20°C			

Finally, twelve acclimation experiments were carried out (Table 2). All the experiments were started at 20°C. Six experiments underwent a temperature variation of ± 5°C at three different TCRs (V2, V3 and V4) and six additional experiments experienced a temperature variation of ± 9°C at three different TCRs (V2’, V3’ and V4’). Once the corresponding final temperature was achieved in each experiment, *D*. *magna* individuals were exposed to that temperature for the time required to achieve FC stabilization. During the acclimation time, the water was changed daily, and new spirulina suspension was added to analyse the time evolution of the suspended spirulina particle concentration after 4 hours each day and to detect potential acclimation to temperature by *D*. *magna*. Once the filtration was stabilized, *D*. *magna* individuals were re-exposed to the initial long-term acclimation temperature (20°C) for the following days until recovering their initial filtration values. *D*. *magna* survival was evaluated daily and dead individuals were replaced with living ones taken from the reservoir cultures subjected to the same experimental conditions.

**Table 2:** Temperature change rates for acclimation and re-acclimation conditions studied.

V(°C/day)			7 day period	V’(°C/day)			5 day period
5	10	20		4.5	9	18	
20°C →25°C			Acclimation to 25°C	25°C →20°C			Re-acclimation to 20°C
20°C →15°C			Acclimation to 15°C	15°C →20°C			

## Results

**Fig 2:**
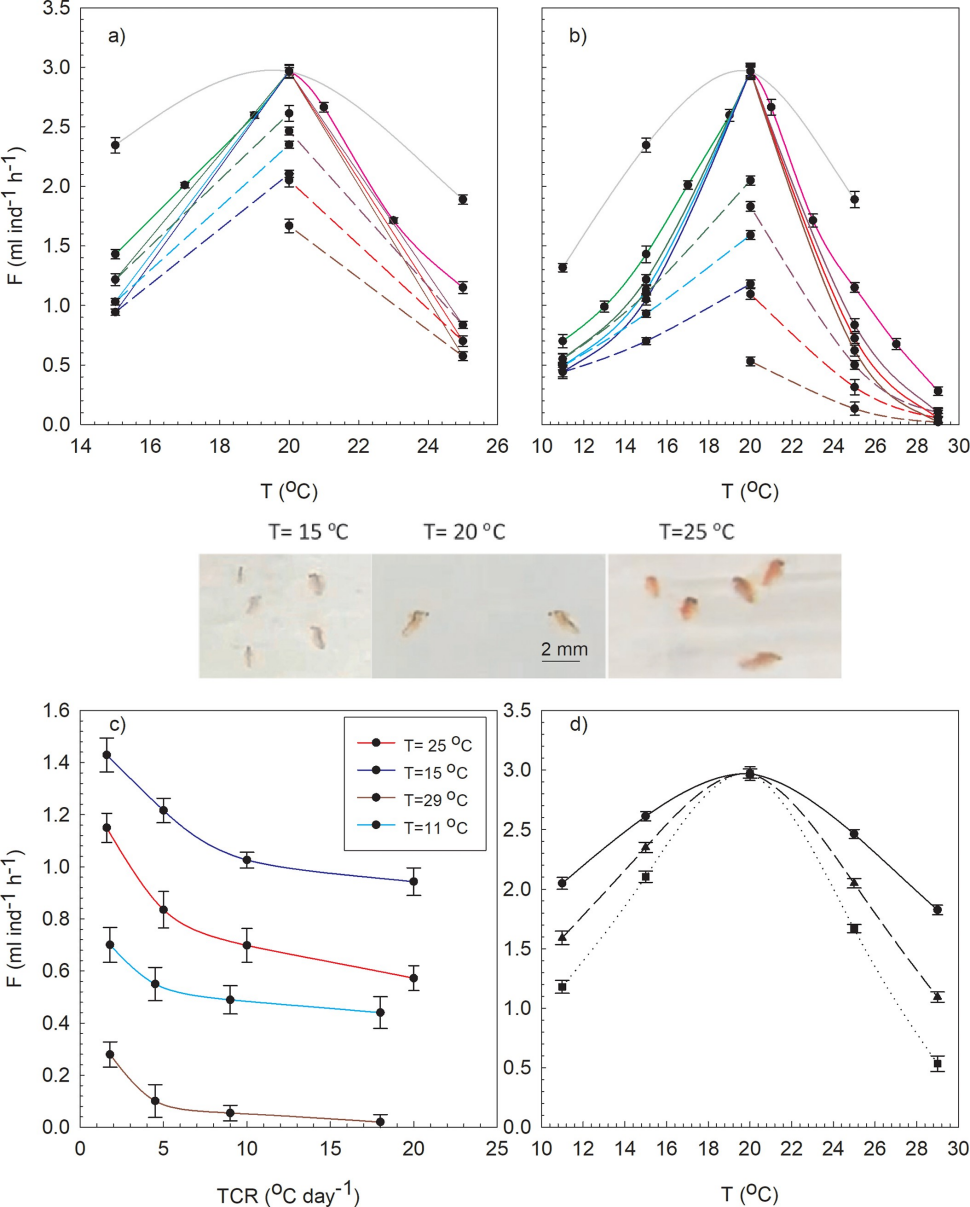
Effects of TCR on FC of *D*. *magna*. A) FC (F, in ml ind^-1^ h^-1^) of *D*. *magna* in the forward (continuous lines), from 20 to 15 or 25°C, and the backward (dashed lines), from 15 or 25°C to 20°C, temperature changes for four TCRs: V1, V2, V3 and V4. The upper continuous grey line corresponds to the FC achieved after acclimation of *D*. *magna* individuals to the corresponding temperature. B) FC (F, in ml ind^-1^ h^-1^) of D. magna in the forward (continuous lines), from 20 to 11 or 29°C, and the backward (dashed lines), from 11 or 29°C to 20°C, temperature changes for four TCRs: V1’, V2’, V3’ and V4’. The upper continuous grey line corresponds to the FC achieved after acclimation of D. magna individuals to the corresponding temperature. C): FC (F, in ml ind^-1^ h^-1^) of D. magna as a function of TCR (°C/day) for the different temperatures and for the analysed TCRs: V1, V2, V3, V4, for 15°C and 25°C and V1’, V2’, V3’, V4’, for 11°C and 29°C. D): FC (F, in ml ind^-1^ h^-1^) of *D*. *magna* obtained for the backward temperature changes (re-establishment of the optimal temperature of 20°C) after the different exposures to temperatures of 11, 15, 25 or 29°C, at three TCRs. The attached images are captures from the video recordings taken after 4 days of exposure to three different temperatures (15, 20 and 25°C). In all the figures each point represents the mean filtration capacity, F, for the different replicates and the corresponding standard deviation.

The optimal temperature for *D*. *magna* filtration was found to be 20°C, coincident with the long-term acclimation temperature (Fig 2A and 2B). Both above and below this temperature, FC decreased significantly, but even more so when high temperatures and/or fast temporal changes of temperature were experienced (Fig 2). Therefore, higher TCR led to a greater decrease in short-term FC (Fig 2A, 2B and 2C), even when the initial optimal temperature had been re-established (Fig 2D). At TCRs between 1.6 and 10°C/day, a decreasing trend in the FC was observed, whereas above 10°C/day the FC reached almost constant values, regardless of the TCR (Fig 2C). Moreover, at temperature changes of both ± 5°C and ± 9°C, regardless of the TCR *D*. *magna* individuals were less affected by low temperatures than they were by high ones. Globally, 15°C resulted in being the least unfavourable temperature for *D*. *magna* individuals, whereas 29°C was the most unfavourable and the maximum negative effect, where the FC reached values near to 0 ml ind^-1^ h^-1^, i.e., almost complete inhibition in *D*. *magna* filtration (Fig 2), were observed.

**Fig 3:**
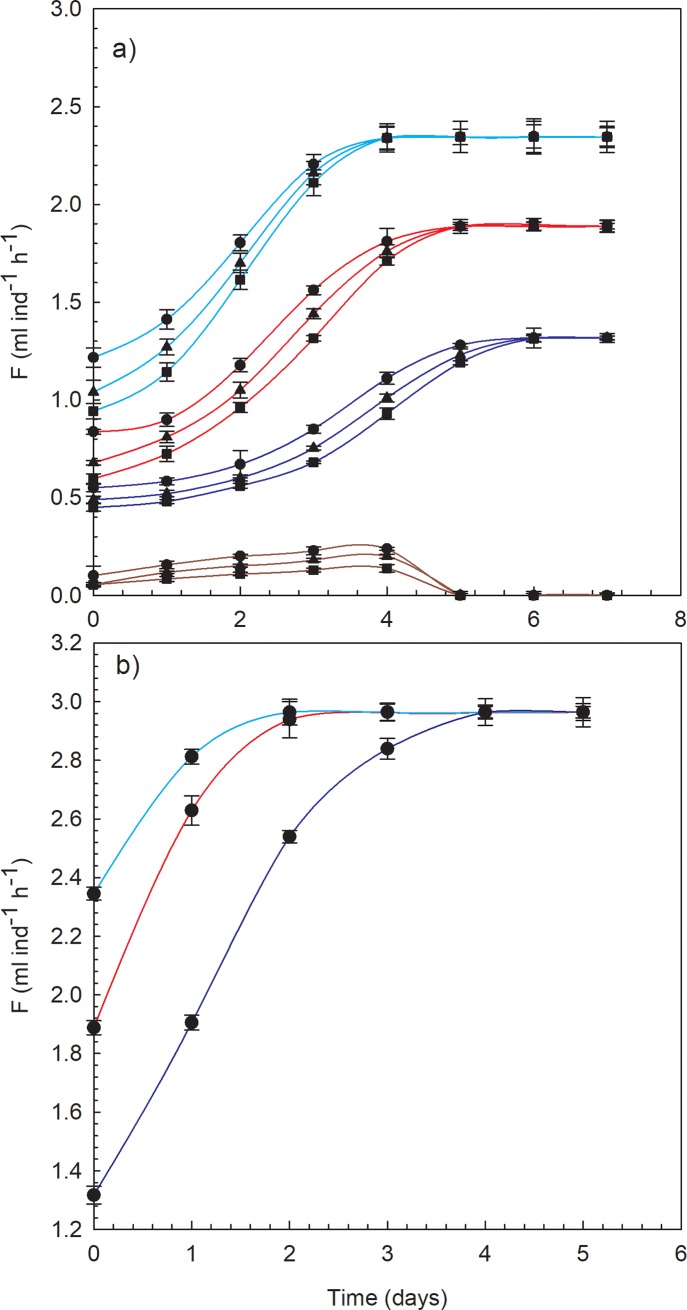
Thermal acclimation of *D*. *magna*. A): FC (F, in ml ind-1 h-1) of *D*. *magna* as a function of the days exposed to the final temperature (light blue lines: 15°C, red lines: 25°C, dark blue lines: 11°C, and brown lines: 29°C) at three TCRs (circles: V2 or V2’, triangles: V3 or V3’, and squares: V4 or V4’). B): FC (F, in ml ind-1 h-1) of D. magna as a function of the re-exposure days to the optimal temperature (20°C), after acclimation to experimental temperatures (light blue line: 15°C, red line: 25°C, and dark blue line: 11°C). In the all figures each point represents the mean filtration capacity, F, for the different replicates and the corresponding standard deviation.

Acclimation experiments showed that, regardless of the TCRs, *D*. *magna* individuals were able to acclimate to a vast range of temperatures, as long as the temperature did not reach the pessimum range (Fig 3A). Time until acclimation was also temperature dependent. Individuals were able to acclimate to 15°C within 4 days, to 25°C within 5 days and to 11°C within 6 days, reaching filtration values significantly above those obtained for the first 4h after the change in temperature (Fig 3A). Acclimation to 29°C was not possible because 100% mortality was reached after 5 days of exposure (Figs A and B in S1 Appendix), thus confirming the adverse effect the pessimum range exerts. Moreover, the final FC of acclimated *D*. *magna* individuals differed between temperatures. It was at its highest at 15°C and its lowest at 11°C, but never again reached the maximum levels seen at 20°C—the optimal temperature—(i.e., quasi-acclimation) (Figs 2A, 2B and 3A). Nonetheless, *D*. *magna* individuals were proven to be capable of recovering their initial FC after 2 days of reacclimation at 20°C for the 15°C and 25°C experiments, and after 4 days for the 11°C experiments (Fig 3B).

## Discussion

Global warming and the associated increase in sudden temperature shifts mean thorough studies into the effects such events have on *D*. *magna* and the responses of their populations should be pursued. Rapid changes in water temperature appear to have direct consequences on the FC of *D*. *magna*. Starting at the temperature of maximal FC (20°C), we observed that over the 4h that followed the temporal temperature change there was a significant decrease (even greater at higher temperatures) in the FC of *D*. *magna*. This effect differed depending on the TCR. For the experiments with temperature changes of ± 5°C, the FC at the highest TCR was ≈9% lower than the FC of the lowest TCR. However, this difference was almost absent for the ± 9°C temperature change experiments, where the FC of the highest TCR was just ≈3% lower than that obtained for the lowest TCR. At 29°C, *D*. *magna* individuals were so severely affected that the short-term FC following the temperature change was almost null and independent of the TCR. This drastic decrease in FC coincided with mortality rates of around 50% in the day following exposure to the final temperature for the highest TCR (Figs A and B in S1 Appendix). High temperature tolerance depends largely on the ability of oxygen transport mechanisms to cover the mounting demand for oxygen associated to the increase in metabolism that follows an increase in temperature [25–28]. Haemoglobin (Hb) is the element central to the oxygen transport system, and is very important for thermal tolerance [52]. This increasing demand for oxygen that follows a temperature increase, along with less oxygen being available because of the reduced solubility in the water, drives the need for an intense synthesis of Hb [10]. This adjustment of Hb is not immediate, so that a sudden rise in temperature leads to a short-term mismatch of oxygen transport and energy demand, which affects the physiological functions of the organisms [26,27] and, therefore, causes a decrease in FC and variations in the swimming speed. We observed that Daphnia individuals tended to stay at the bottom of the containers at the lowest temperature treatments and swam upwards during the highest temperature treatments. We also checked the swimming velocity of the individuals at each temperature treatment but we did not get clear and consistent results, since we observed a high variability in swimming behaviour and velocity amongst individuals. As an example, as individuals became more stressed, the number of loopings and hoppings increased, making it even more difficult to calculate an average swimming speed. Moreover, an excessive increase in temperature means that the individuals enter the upper pessimum range [24], leading to a severe mismatch of oxygen transport and energy demand that causes the transition to anaerobic metabolism and, finally, the collapse of higher physiological functions [25–27,29], thus almost inhibiting the FC of *D*. *magna*, as well as the size growing of individuals. Exceeding the pessimum range limit or the extent of exposure to it, leads to death [24,26,27]. This limit was found to be exceeded at 29°C. In cold water conditions, oxygen demand is reduced, and oxygen availability is high thanks to the increased solubility in water. However, the lower temperature limit, indicating the beginning of the lower pessimum range [24], is characterized by the cessation of ventilation and perfusion and the muscular activity needed for the correct supply of oxygen [29]. Subsequently, PO_2_ in the blood diminishes [25] and anaerobic metabolism is activated [27]. Although results show that 11°C was highly unfavourable to individuals, survival was still above 70%, thus indicating that the lower critical temperature limit had yet to be reached. Therefore, it would appear that *D*. *magna* individuals are more vulnerable to an increase, rather than a decrease, in temperature, even when the magnitude of the temperature change is the same.

Although the decrease in FC was more significant with the increase in TCR, this decreasing trend was almost absent at TCRs above 10°C/day. This results would suggest that at low TCRs, the activation of responses, for instance changes in the expression of some heat shock proteins [40] or haemoglobins [10,38] or changes in the anaerobic capacity of mitochondria [53,54], occur on the same temporal scale as the temperature change, so that the advantages resulting from that response are already distinguishable in short-term measurements. However, at TCRs above 10°C/day, the activation of the temperature response mechanisms may still not be noticeable, so that the resulting FC values are almost independent of the TCR. Subsequently, results show that the temperature changes *D*. *magna* individuals must cope with during DVM are associated with metabolic costs that greatly depend on the temperature gradient and, albeit to a lesser extent, on the magnitude of TCR. Thus, the longer and intensified summer thermal stratification caused by global warming [49] the greater the impact on *D*. *magna* populations performing DVM. However, if the temperature gradient becomes too high, *D*. *magna* individuals will most probably avoid DVM to avoid extreme temperature changes [55], thus making them more exposed to predation.

When reverting the temperature change applied at the same TCR, *D*. *magna* individuals were able to recover quickly, such that the FC increased again. These results match those obtained by Paul et al. [25], who observed that when exposing *D*. *magna* individuals acclimated to different temperatures (10, 20 or 30°C) to 35 or 36°C there were significant differences in LT_50_ (temperature of 50% mortality) directly after heat exposure, but these differences had almost disappeared after 24h of reincubation to the initial temperature, confirming their ability to quickly recover from thermal stress. However, in the present study, during the first 4h of re-exposure to the long-term acclimation temperature, FC was still below the initial value, and was more negatively affected by higher TCRs, as well as higher final temperatures. Activating thermal response mechanisms takes some time. Likewise, then, recovering the initial metabolic conditions also needs its time, so that even when reverting to optimal conditions, short-term deficiencies in the FC are observed.

Gienapp et al. [56] highlighted the importance of analysing the capacity of organisms to perform microevolutionary responses to rapid climate change, and made a clear distinction between genetic and phenotypic responses. Following this line of study, Van Doorslaer et al. [57] demonstrated the enormous capacity *D*. *magna* have to adapt, both by genetic adaptation and plasticity, to temperature changes within a short time frame. The strong phenotypic plasticity featured by *D*. *magna* is explained by their ability to regulate certain genes in response to sudden and drastic temperature shifts [38,40]. This ability to adapt to a constantly changing environment is due to the enormous evolutionary potential *D*. *magna* has as a result of its great genetic variability [58]. A key factor in acclimating to a certain temperature is the ability to adjust the synthesis of Hb [10]. The time needed to acclimate to warm temperatures is similar to that required for haemoglobin induction, with any major changes occurring during the first three days [29]. Moreover, acclimating to cold temperatures is usually achieved by increasing the aerobic capacity of the mitochondria [53,54]. Although sudden temperature changes have been proven to have decisive short-term effects on the metabolism of *D*. *magna*, acclimation experiments have confirmed that, over time, these organisms are able to adapt to certain temperatures, regardless of the speed of the change in temperature. In our experiments, quasi-acclimation occurred in 4 days (15°C experiments), 5 days (25°C experiments) and 6 days (11°C), after which FC reached values almost three-fold higher than those obtained during the first 4h of exposure to the final temperature. These results agree with Precht [59], who found signs of thermal acclimation by *D*. *magna*, in terms of heart rate and leg movements, after 5–7 days. However, the acclimation was temperature-dependent and is probably highly influenced by the initial acclimation temperature (20°C), such that the final FC was highest for 15°C and lowest for 11°C. Acclimation was not possible at 29°C, since all *D*. *magna* individuals died after 5 days of exposure to that temperature. These results confirm that 29°C is within the upper pessimum range for *D*. *magna*, allowing only short-term survival [25]. However, we did not reach the lower pessimum range, since our results suggest that 11°C is still within the suboptimal temperature range. These observations are in accordance with Chen et al. [60], who found that after 24h of exposure at 10°C there were still no significant changes in the motility strength of *D*. *magna*, in comparison to 20°C. Intensified synthesis of Hb at high temperature experiments was confirmed by the colour of *D*. *magna* individuals. At 25°C, they were crimson and then lost coloration as the temperature was reduced, until they were almost transparent at 11°C. Although at 29°C Hb synthesis should also have increased, the absence of coloration after 4 days of exposure to that temperature confirmed that *D*. *magna* individuals were unable to acclimate to that temperature. In other words, sudden temperature changes initially cause a decrease in FC but, once the regulatory machinery has been adjusted to the final temperature, *D*. *magna* individuals are able to recover in great measure (quasi-acclimation), adapting their metabolism to the new conditions within a few days, albeit as long as the conditions remain within their tolerable ranges.

Even after *D*. *magna* individuals had acclimated to the corresponding final temperature, organisms were able to recover the maximal FC within 2–4 days of reacclimation to 20°C. Therefore, even though *D*. *magna* individuals quickly adjust their regulatory machinery to sudden temperature changes, these responses are reversible, so that their metabolism is able to recover easily from such events when the initial long-term acclimation temperature is re-established. However, mortality analysis (Figs A and B in S1 Appendix) show that the survival of *D*. *magna* individuals decreases significantly as a function of the speed of the temperature change, so that even though individuals seem to be able to recover easily in terms of metabolism, these temperature change events come at a high cost.

Furthermore, depending on the origin of the *D*. *magna* populations, there is a great variability in their tolerance to temperature [38]. The *D*. *magna* cultures used for this study originally came from a water sanitation system located in the north-east of Spain. Thus, the populations had adapted to a typical Mediterranean climate. The average air temperature (AAT) of the hottest month (July) over the period of 1971–2017 in this area was 23.3°C. However, in the last two decades there has been a temperature increase of 0.4°C/decade, so that in the last 10 years the AAT in July has been 24.1°C [61]. This fact must be taken into account when interpreting the results obtained, since local natural selection provides *D*. *magna* populations with more or less temperature tolerance, depending on their needs [38,62]. Moreover, among *Daphnia* species there are significant differences in thermal tolerance which depend on the thermal characteristics of their habitat. In other words, *D*. *magna* are quite a heat tolerant species [10,63]. Furthermore, differences in the performance at high or low temperatures can also occur, depending on whether individuals belong to winter or summer clones [64]. Finally, the preferred temperature and the thermal tolerance window for *D*. *magna* are both directly influenced by the acclimation temperature [25,29,30], although the upper thermal limit is hardly changed by temperature acclimation [25,29]. Therefore, *D*. *magna* individuals present the highest swimming activity and lowest lactate production (anaerobic metabolism) near to their acclimation temperature, showing that *D*. *magna* swimming depends on the aerobic provision of energy and is therefore maximised in the optimal range of thermal tolerance [25,29], as is their FC. All these facts were considered when interpreting and comparing the results obtained, as they all directly influence *D*. *magna’s* thermal tolerance and temperature preference.

## Supporting information

S1 Appendix This appendix file contains additional information on both the calculation of *D. magna* filtration capacity and the survival of *D. magna* for experiments when changing temperatures. (DOCX)

S1 Dataset This dataset file contains all the data obtained from all experiments with results plotted in Figs 2 and 3, as well on Figs A and B in the S1 Appendix file. (XLSX)
