# Supplementary material for: Temperature-driven response reversibility and short-term quasi-acclimation of Daphnia magna
Source: PLoS One. 2018 Dec 21;13(12):e0209705. doi: 10.1371/journal.pone.0209705 (PMC6303020; doi:10.1371/journal.pone.0209705)
Supplement: S1 Appendix — (DOCX) [file pone.0209705.s001.docx]

1. ***Calculating D. magna filtration capacity***

A laser particle size analyser, the Lisst-100x (Sequoia Inc.), was used to measure the spirulina particle size distribution in the suspension. Pau et al. [4] studied particle removal efficiency by *Daphnia magna* and showed that particle concentration by *D. magna* ingestion decreased exponentially. They found that after approximately 4 hours, the initial concentration decreased by e^−1^ = 0.37 and so fixed this as the characteristic time in all the experiments. Therefore, samples from each experiment were taken at t_0_ (moment when the acclimation period was completed, and new spirulina suspension was added) and at t_4,_ to analyse the time evolution of the suspended spirulina particle concentration. The Lisst instrument consists of multi-parameter measurements taken through scattering at a set of multiple angles. The particles are illuminated by a laser beam formed by collimating the output of a diode laser. The focal plane of a receiving lens detects the scattering by particles. To distinguish the particle size, logarithmically distributed in the range of 2.5 – 500 μm, a detector consisting of 32 rings is placed in the focal plane. Each ring measures the scattering at a particular small range of angles and this energy distribution sensed by the ring detector is recorded by the Lisst instrument. After the data collection is completed, the data are offloaded and mathematically inverted to obtain the size distribution using the absolute volume concentration of each particle size. Particle concentration was calculated by integrating the concentration of the particles within the *D. magna* feeding range, which is between 2.5 and 30 μm in diameter [31]. Therefore, the volume concentration of particles within the range of 2.5 to 30 μm was used to evaluate particle removal.

By considering the exponential decrease of the suspended particle concentration, the concentration can be described by the following equation:

$c=c_{0}e^{-kt}$

where k is the rate of particle removal by both sedimentation (k_s_) and *D. magna* filtration (k_Dph_), i. e. $k=k_{s}+k_{\mathrm{Dph}}$. Therefore, it is possible to write k as

$k= -\frac{1}{t}ln\left( \frac{c}{c_{0}} \right)$

and k_s_ can be fixed from the control experiment without *D. magna* individuals (where k_Dph_ = 0). k_Dph_ was calculated for the rest of the experiments. Moreover, the rate of particle removal by *D. magna* filtration depended on the filtering rate of each *D. magna* individual (F, in mL ind^-1^ L^-1^) and the *D. magna* concentration, so that [4]

$k_{Dph}=F x C_{Dph}$

1. ***Survival of D. magna in changing temperatures***

Mortality analysis were carried out for every experiment by counting the surviving individuals after 24h of exposure to the final temperature and then every day for the acclimation experiments.

Results show that the survival decreased as a function of the TCR (Fig A). However, a significant difference was observed depending on the temperature. For temperature changes of ± 5 °C, short-term survival at V4 was ≈ 20 % lower than survival at V1, with the survival values at 15 °C being ≈ 0.5 % higher than those at 25 °C. In contrast, at temperature changes of ± 9 °C, mortality at V4 was ≈ 30 % (11 °C experiments) and ≈ 40 % (29 °C experiments) higher than at V1, proving that such significant temperature changes have extremely negative effects on the viability of *D. magna*.

Acclimation experiments showed that the survival over time of individuals was almost not affected by exposure to 15 °C. However, at 25 °C and 11 °C mortality started to increase, so that after 6 days of exposure it was ≈ 10% higher at 25 °C and ≈ 20% higher at 11 °C. Finally, 29 °C proved to be lethal for *D. magna*, thus causing a sharp drop in survival over time, reaching 100 % mortality after 5 days of exposure (Fig B).


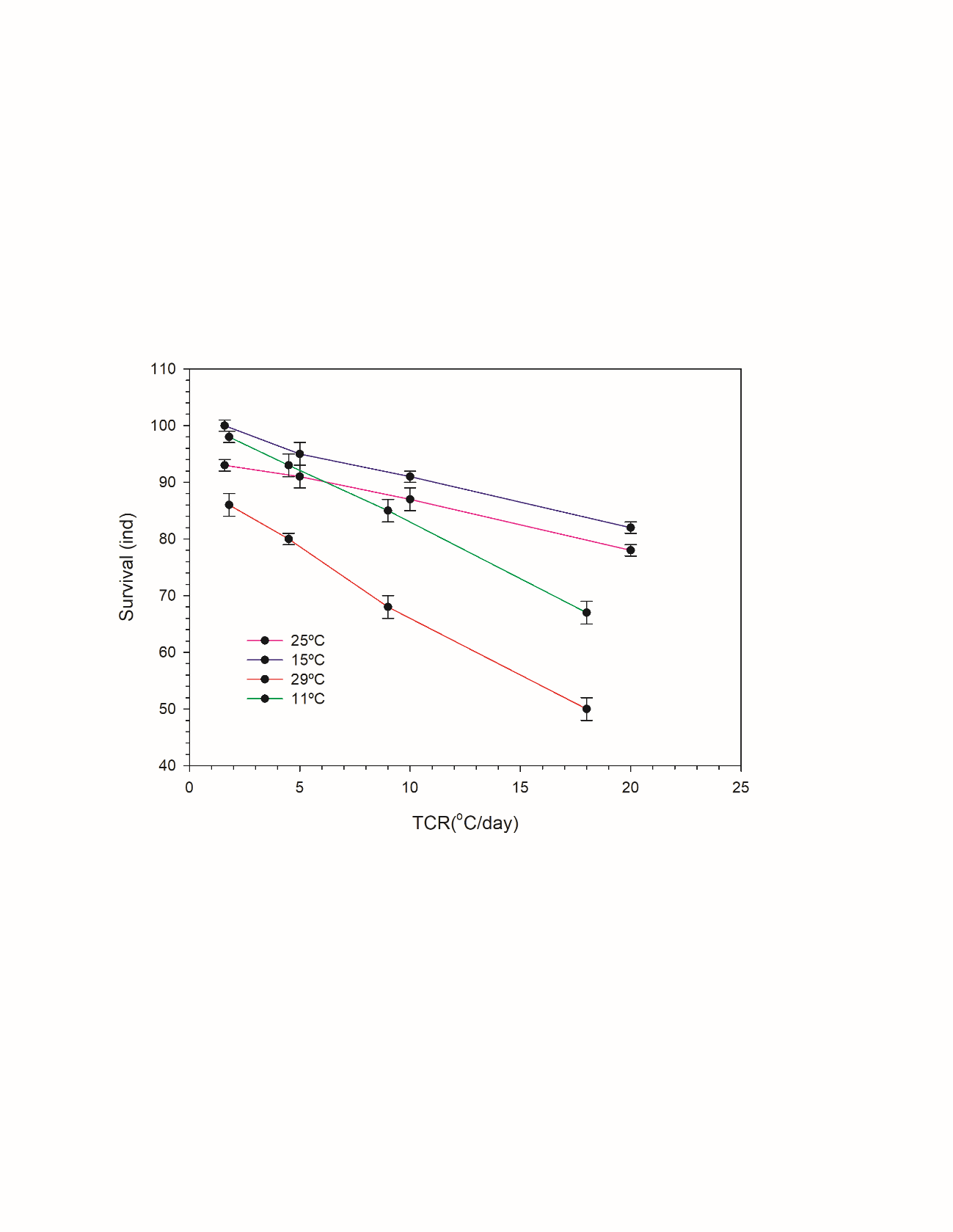


**Fig A**. Survival of *D. magna* as a function of TCR (°C/day) for the four different temperature exposures (11, 15, 25 and 29 °C).


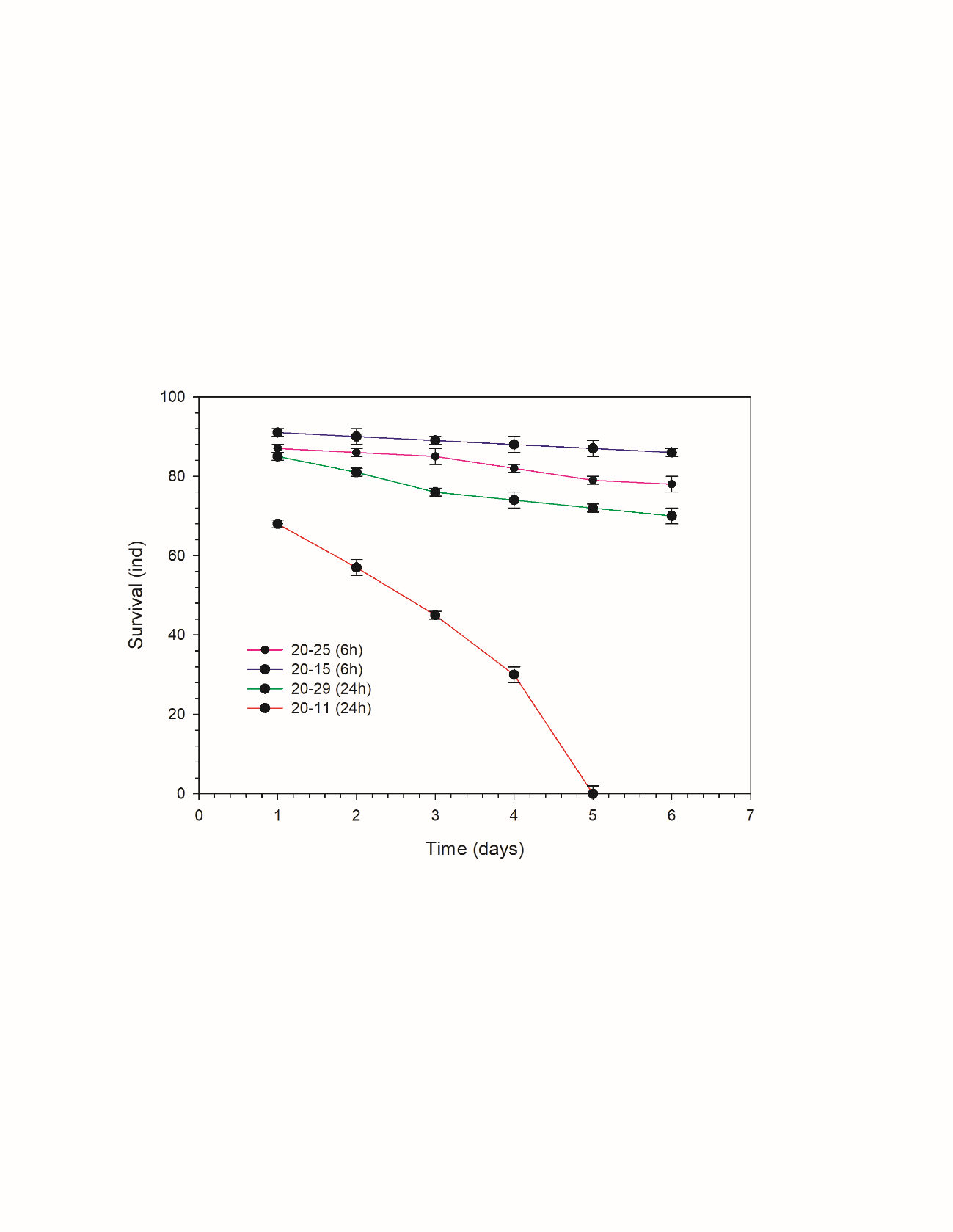


**Fig B**. Survival of *D. magna* as a function of exposure time (days) to the four different temperatures (11, 15, 25 and 29 °C).
